# Reliability of the universal goniometer for assessing active cervical range of motion in asymptomatic healthy persons

**DOI:** 10.12669/pjms.322.8747

**Published:** 2016

**Authors:** Muhammad Nazim Farooq, Mohammad A. Mohseni Bandpei, Mudassar Ali, Ghazanfar Ali Khan

**Affiliations:** 1 Muhammad Nazim Farooq, PhD Student. University Institute of Physical Therapy, Faculty of Allied Health Sciences, University of Lahore, Lahore. Margalla Institute of Health Sciences Rawalpindi, Pakistan; 2 Prof. Dr. Mohammad A. Mohseni Bandpei, PhD. Visiting Professor, University Institute of Physical Therapy, Faculty of Allied Health Sciences, University of Lahore, Lahore, Pakistan. Iranian Research Centre on Aging, Department of Physiotherapy, University of Social Welfare and Rehabilitation Sciences, Evin, Tehran, Iran; 3 Mudassar Ali, PP-DPT. Margalla Institute of Health Sciences, Rawalpindi, Pakistan; 4 Ghazanfar Ali Khan, MS-OMPT. Margalla Institute of Health Sciences, Rawalpindi, Pakistan

**Keywords:** Universal goniometry, Neck, Range of motion, Reliability

## Abstract

**Objective:**

To determine within-rater and between-rater reliability of the universal goniometer (UG) for measuring active cervical range of motion (ACROM) in asymptomatic healthy subjects.

**Methods:**

Nineteen healthy subjects were tested in an identical seated position. Two raters used UG to measure active cervical movements of flexion, extension, right side flexion, left side flexion, right rotation and left rotation. Each motion was measured twice by each of the two raters and was re-measured all over again after one week. Data analysis was performed using the intraclass correlation coefficient (ICC).

**Results:**

The results demonstrated excellent within-session (ICC2,1 = 0.83 to 0.98) and between-session (ICC2,2 = 0.79 to 0.97) intra-rater reliability and excellent inter-rater reliability (ICC2,2 = 0.79 to 0.92).

**Conclusion:**

Considering above results it is concluded that UG is a reliable tool for assessing ACROM in a clinical setting for healthy subjects.

## INTRODUCTION

Disorders of the cervical spine are the leading health problems and significant cause of disability.^1^-^3^ Normal cervical spine range of motion (ROM) is usually altered by these disorders.^4^, ^5^ Cervical ROM is frequently measured when patients having neck pain visit the physical therapy clinics.^6^ It may help the physiotherapist to determine the diagnosis, formulate the prognosis, design the plan of care, check the progress, and evaluate the efficacy of treatment.^4^, ^7^-^9^ Objective measurements aid in maintaining the patient’s interest by keeping him informed about the improvement.^8^

Various measurement devices are available for cervical ROM evaluation,^7^, ^10^ going from simple instruments like universal goniometer (UG) to electromagnetic 3D Fastrak measurement system or 3D ultrasound equipment.^7^, ^11^-^13^ But these equipment are not very accessible for clinical practice due to high cost and complexity for use in specific segments. Therefore, it is very important to consider about the reliability, easiness to use and the cost of the device while choosing the most suitable measurement device.^5^, ^8^, ^9^ UG, a tool used most commonly for evaluating joint ROM in the clinical settings,^7^, ^14^, ^15^ comes out as a simple alternative for global use at low price.

Unfortunately, clinical measurements of the cervical ROM by UG are often inconsistent and are claimed to be less precise compared to the measurements of other body’s joints mobility.^7^, ^16^ These inconsistencies and lack of accuracy may be due to inter-tester differences in measurement technique, including differences in patient and goniometric positioning.^4^, ^7^, ^17^ This may also be due to improper alignment of the goniometer, lack of anatomical landmarks, variability of the neutral head position and soft tissues thickness in the cervical region.^17^, ^18^

Many studies have been published on the inter-tester and intra-tester reliability of the UG. Several authors have found greater intra-tester reliability than inter-tester reliability^16^, ^17^, ^19^ in both clinical and research settings. Yankai & Manosan,^15^ however, found both inter-tester and intra-tester reliability high. Chaves et al.^17^ on the other hand, found moderate intra-tester and low to moderate inter-tester reliability.

Moreover, in the previous studies there were lack of qualified and experienced personnel, and standardized protocols for measurement and for performance of the motions which make it difficult to compare and use the data produced by these studies.^15^, ^17^ The present study was performed to evaluate the intra-rater and inter-rater reliability of the UG for active cervical range of motion (ACROM) measurements on normal healthy subjects.

## METHODS

This is a double-blind inter and intra-rater reliability study. A relatively homogeneous study group consisting of 19 healthy subjects (10 male and 9 female) between 20 to 24 years of age were recruited from students of Margalla Institute of Health Sciences Rawalpindi following power calculation based on the work of Walter et al.^20^ Demographic characteristics of participants are summarized in Table-I. All the participants were without neck pain at the time of measurement and had no previous history of neck pain or cervical spine pathology. Participants having cervical dysfunction, trauma, surgery or neurological disease were excluded.

**Table-I:** Physical characteristics of the subjects (N = 19).

Characteristic	Mean	SD	Range
Age (years)	21.32	1.29	20-24
Height (cm)	168	5.62	157-175
Weight (kg)	62	8.06	45-80
BMI	21.95	2.52	18-27.7

The study was performed at the Physiotherapy department of Margalla Institute of Health Sciences Rawalpindi. The study was approved by the Ethics Committee of University of Lahore. All prospective subjects were provided with written information regarding the purpose and nature of the study by information sheet and were given the opportunity to ask any question they may have. A consent form was signed by each subject prior to study.

### Blinding

Each rater was blinded to the other rater’s findings. Each subject was also blinded to the results.

### Equipment

A large UG having 12-inch arms and full-circle plastic body was used for measuring ACROM. It is commonly used to measure joint ROM in clinics.^14^, ^15^

### Raters

Two raters, trained in the measurements of joints ROM, measured ACROM with UG in all subjects. Both raters were qualified physiotherapists having more than eight years of clinical experience. An additional Physiotherapist read and recorded all measurements values to reduce bias.

### Measurement procedure

Neck ROM was measured in a standardized sitting position to remove errors and movement compensation.^15^ The subjects were asked for sitting with their back straight and strapped to the back of the wooden chair. Subject’s ankles, knees and hips were positioned at right angle and arms were folded across the chest to minimize thoracic movement.

Measurements were taken on two different sessions with one week period. During each session, every subject was assessed twice by each of the two raters in a set sequence of six ACROM. The first movement was flexion, second extension, third right side flexion, fourth left side flexion, fifth right rotation and at the end left rotation. All these cervical movements were performed in the same order during each measurement across both test sessions. Prior to testing, each subject was requested to perform all the six cervical motions to end range actively in the set sequence to reduce creep and to familiarize him with the testing procedure.^10^, ^15^ Same and uniform verbal instructions were given to all subjects to take these measurements.

The examiners were randomly assigned for the measurement order. Both the examiners were alternated with each other to become the first examiner. The first examiner positioned each subject and provided him the verbal instructions about the performance of set sequence of movements. Following the instructions, all the six cervical movements were measured by the first examiner in the set sequence, and then all these movements were repeated and measured again. After recording all the measurements, the first examiner departed the room and the second examiner entered the room and performed the measurements with same protocol. Five minutes rest was given to subjects between these trials of measurements. In order to reduce bias, the examiners did not look at each other while taking measurements.^15^ All the subjects were measured again by both examiners after one week period in the same way as described before, although the rater position was changed by the examiners.

### Data analysis

Data analysis was performed using SPSS 21 version. Descriptive statistics for goniometric measurements of ACROM for each movement were calculated using mean and standard deviations. Intraclass correlation coefficient (ICC) and 95% confidence intervals (CIs) were computed to find out the reliability of the measurements taken by UG.^21^ ICC is used to determine the size and trend of correlation between the variables.^22^ In addition, standard error of measurement (SEM) was computed using the formula SD × √ (1 – ICC), where SD represents standard deviation and ICC is the reliability coefficient for the particular measurement.^21^ Within session intra-rater reliability (ICC2,1) was determined by comparing the two measurements of each cervical movement acquired by each examiner on each test session. Between-session intra-rater reliability (ICC2,2) was calculated by comparing the average of two measurements of each movement recorded by each examiner on each test session. For within-session inter-rater reliability (ICC2,2), the averages of two measurements of each cervical movement recorded by each examiner on each test session were compared.

There are different guidelines to interpret ICC, but one most reasonable criteria is as follow; ICC value < 0.4 indicates poor reproducibility, ICC values from 0.4 to 0.75 show fair to good reproducibility, and ICC ≥ 0.75 indicates excellent reproducibility.^23^, ^24^

## RESULTS

Descriptive statistics for measuring ACROM using the UG are shown in Table-II. The mean of each cervical movement recorded by both the examiners was similar between sessions and raters.

**Table-II:** Cervical ROM values* obtained using the goniometer (N = 19).

Movement	Examiner 1				Examiner 2			

	Session 1		Session 2		Session 1		Session 2	

	1^st^ Assessment	2^nd^ Assessment	1^st^ Assessment	2^nd^ Assessment	1^st^ Assessment	2^nd^ Assessment	1^st^ Assessment	2^nd^ Assessment

	Mean±SD	Mean±SD	Mean±SD	Mean±SD	Mean±SD	Mean±SD	Mean±SD	Mean±SD
Flexion	45.90±9.05	46.79±9.18	44.47±7.21	46.16±7.21	45.05±7.23	44.90±7.06	44.26±5.52	45.74±6.87
Extension	47.16±7.38	47.42±7.81	46.95±8.48	48.63±8.79	43.63±6.13	44.32±5.71	42.74±4.42	43.68±4.88
Right side flexion	34.74±6.83	34.95±7.41	32.47±5.40	33.63±6.69	31.53±6.19	31.95±7.39	31.68±5.66	31.74±6.60
Left side flexion	34.79±6.40	36.58±5.98	35.21±5.37	35.68±5.46	31.37±4.52	32.79±6.08	31.53±5.78	32.84±5.51
Right rotation	64.63±5.17	65.32±4.75	63.32±6.35	64.47±6.51	66.47±5.98	67.26±6.03	65.63±7.20	67.21±6.84
Left rotation	65.79±4.04	67.68±3.40	66.21±4.33	67.42±4.05	65.74±5.62	67.63±4.98	66.63±5.61	67.84±4.88

### Intra-rater reliability

According to the criteria suggested by Fleiss^23^ and Rosner,^24^ excellent within-session intra-rater reliability was observed for both raters measuring ACROM using UG, with ICC (2,1) ranging from 0.83 to 0.98. The results also found excellent between-session intra-rater reliability, with ICC (2,2) ranging from 0.79 to 0.97.

The SEM for all movements varied from 0.90 to 2.62 degrees for within-session intra-rater reliability, and 0.87 to 2.36 degrees for between-session intra-rater reliability. The values of ICC and SEM are presented in Table-III.

**Table-III:** Intra-rater reliability for the two raters to test active cervical ROM using the goniometer.

Movement	Rater	Within Session				Between Session	

		Session I		Session II			

		ICC (95% CI)	SEM (deg)	ICC (95% CI)	SEM (deg)	ICC (95% CI)	SEM (deg)
Flexion	R1	0.96 (0.90-0.99)	1.81	0.97 (0.92-0.99)	1.24	0.96 (0.88-0.98)	1.59
	R2	0.88 (0.71-0.95)	2.40	0.87 (0.70-0.95)	2.17	0.91 (0.75-0.96)	1.86
Extension	R1	0.95 (0.87-0.98)	1.68	0.91 (0.78-0.96)	2.53	0.93 (0.81-0.97)	2.04
	R2	0.91 (0.79-0.97)	1.74	0.91 (0.79-0.97)	1.37	0.87 (0.67-0.95)	1.77
Right side flexion	R1	0.98 (0.95-0.99)	1	0.92 (0.82-0.97)	1.69	0.94 (0.83-0.98)	1.55
	R2	0.84 (0.64-0.94)	2.62	0.82 (0.59-0.93)	2.49	0.95 (0.86-0.98)	1.35
Left side flexion	R1	0.96 (0.90-0.98)	1.23	0.94 (0.85-0.98)	1.31	0.90 (0.75-0.96)	1.73
	R2	0.88 (0.71-0.95)	1.80	0.83 (0.61-0.93)	2.23	0.94 (0.83-0.98)	1.26
Right rotation	R1	0.86 (0.68-0.95)	1.79	0.98 (0.94-0.99)	0.90	0.79 (0.46-0.92)	2.36
	R2	0.95 (0.88-0.98)	1.32	0.89 (0.74-0.96)	2.26	0.93 (0.81-0.97)	1.63
Left rotation	R1	0.85 (0.66-0.94)	1.42	0.95 (0.87-0.98)	0.92	0.85 (0.62-0.94)	1.41
	R2	0.89 (0.74-0.96)	1.70	0.90 (0.75-0.96)	1.62	0.97 (0.91-0.99)	0.87

ROM = Range of motion, R1 = Rater 1, R2 = Rater 2, ICC = Intraclass correlation coefficient, CI = Confidence interval, SEM = Standard error of measurement, deg = degrees.

### Inter-rater reliability

Within-session inter-rater reliability was excellent for measuring ACROM using universal goniometer, with ICC (2,2) varying from 0.79 to 0.92. The SEM for all movements ranged from 1.40 to 3.35 degrees. The complete results are presented in Table-IV.

**Table-IV:** Inter-rater reliability between the two raters to test active cervical ROM using the goniometer.

Movement	Inter-rater Reliability			

	Session I		Session II	

	ICC (95% CI)	SEM (deg)	ICC (95% CI)	SEM (deg)
Flexion	0.79 (0.46-0.92)	3.35	0.92 (0.78-0.97)	1.80
Extension	0.92 (0.79-0.97)	1.83	0.79 (0.46-0.92)	2.83
Right side flexion	0.89 (0.71-0.96)	2.14	0.87 (0.65-0.95)	2
Left side flexion	0.89 (0.73-0.96)	1.79	0.87 (0.67-0.95)	1.82
Right rotation	0.90 (0.75-0.96)	1.62	0.88 (0.69-0.95)	2.17
Left rotation	0.83 (0.57-0.94)	1.70	0.90 (0.74-0.96)	1.40

ROM = Range of motion, ICC = Intraclass correlation coefficient, CI = Confidence interval, SEM = Standard error of measurement, deg = degrees.

## DISCUSSION

This study was performed to examine the reliability of active cervical movements measured by UG which is commonly used by clinicians. The results demonstrated excellent inter and intra-rater reliability of the UG for measuring active ROM of all cervical movements in healthy subjects.

The authors found the results to be similar to those of Yankai & Manosan,^15^ who demonstrated that intra-rater and inter-rater reliability were high to very high. They found intra-rater reliability ranging from .80 to 0.99 and inter-rater reliability varying from 0.71 to 0.94 when healthy volunteers were measured with UG.

The results of this study are also in agreement with Whitcroft et al.^25^ who compared the accuracy and reliability of UG, tape measurement and visual estimation with CROM device for testing ACROM in healthy subjects. They found considerable high reliability for UG and concluded that the measurement of cervical movements by UG is the most reliable technique when the measurements are taken by aligning goniometer on fixed landmarks.

The findings of the present study, on the other hand, differs from the results of Chaves et al.^17^ who found moderate intra-tester reliability (ICC = 0.43 to 0.54), and poor to moderate inter-tester reliability (ICC = -0.07 to 0.60). As this study was conducted on children, therefore poor procedural understanding and less collaboration may contribute towards lowering the goniometer reliability.

Standard measurement errors in the range of 0.87–2.62 degrees for the intra-rater trials, and 1.40–3.35 degrees for the inter-rater trials highlighted minimal variation in the precision associated with the cervical ROM testing with UG in healthy subjects.

### Strengths and limitations

First, both the examiners were qualified physiotherapists having more than eight years of clinical and teaching experience and remained involved in teaching goniometry to students which may help in increasing the reliability of UG for measuring neck movements. Second, random errors have been minimized by standardizing the procedures. This was accomplished by stabilizing all the subjects to avoid movement compensation and by providing them with the same instructions before measurement. In addition to that an identical environment e.g. same chair, same participant’s orientation, same room, etc. was used to collect the data.

Finally, all the cervical movements were measured with UG in the same set sequence. Therefore, if repetitions increase the range of any movement, it would be identical for all the subjects and would not affect the study results.

The collection of the data on healthy subjects was a limitation of this study which may limit the external validity. Thus, the generalizability of the results of the current study to a patient population may be limited. Because the purpose of current study was to explore the UG reliability, therefore, it seems that an available sample of healthy students was appropriate.

## CONCLUSION

This study found excellent inter and intra-rater reliability of the UG measurements for assessing ACROM. The results established that UG is a reliable device to evaluate cervical ROM. The study proposes that uniform instructions and arrangements for measuring ACROM would contribute towards reducing variations between the examiners and increasing the reliability. The UG is cheap, easy to use, popular instrument and requires minimum training.
